# Supplementary material for: Museum specimens of a landlocked pinniped reveal recent loss of genetic diversity and unexpected population connections
Source: Ecol Evol. 2023 Jan 18;13(1):e9720. doi: 10.1002/ece3.9720 (PMC9849707; doi:10.1002/ece3.9720)
Supplement: Supplementary file 1 — Figure S1. [file ECE3-13-e9720-s001.pdf]

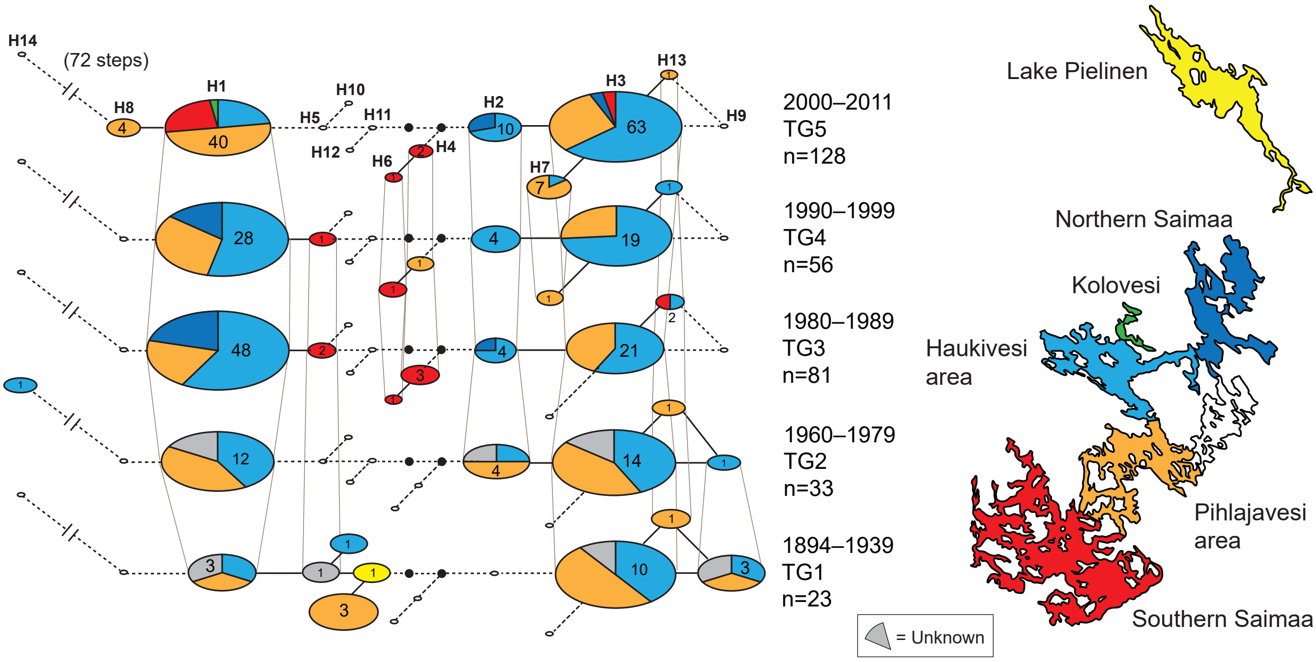

Figure S1. Haplotype network of Saimaa ringed seals during five different time intervals spanning periods from 1894 to the present. In each network, circle size is proportional to haplotype frequency, and sector colors denote the proportion of samples belonging to each of the sampling areas indicated in the map to the right of the networks.
