# Supplementary material for: Museum specimens of a landlocked pinniped reveal recent loss of genetic diversity and unexpected population connections
Source: Ecol Evol. 2023 Jan 18;13(1):e9720. doi: 10.1002/ece3.9720 (PMC9849707; doi:10.1002/ece3.9720)
Supplement: Supplementary file 2 — Figure S2. [file ECE3-13-e9720-s005.pdf]

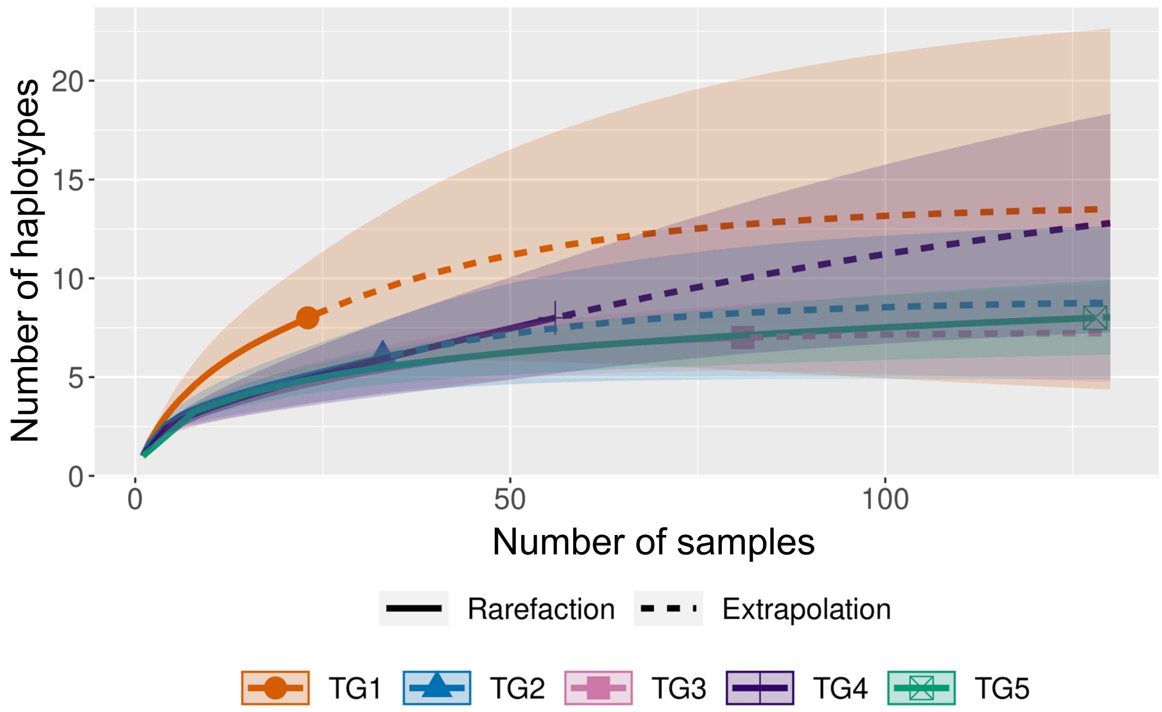

Figure S2. Sample-size-based rarefaction and extrapolation curves for the number of haplotypes in each of the five time periods (TG1 1894–1939 (*N* = 23), TG2 1960–1979 (*N* = 33), TG3 1980–1989 *(N* = 81), TG4 1990–1999 (*N* = 56), and TG5 2000–2011 *(N* = 128)). The shaded regions surrounding lines represent period-specific 95% confidence intervals (see legend).
