# Supplementary material for: Museum specimens of a landlocked pinniped reveal recent loss of genetic diversity and unexpected population connections
Source: Ecol Evol. 2023 Jan 18;13(1):e9720. doi: 10.1002/ece3.9720 (PMC9849707; doi:10.1002/ece3.9720)
Supplement: Supplementary file 3 — Figure S3. [file ECE3-13-e9720-s003.pdf]

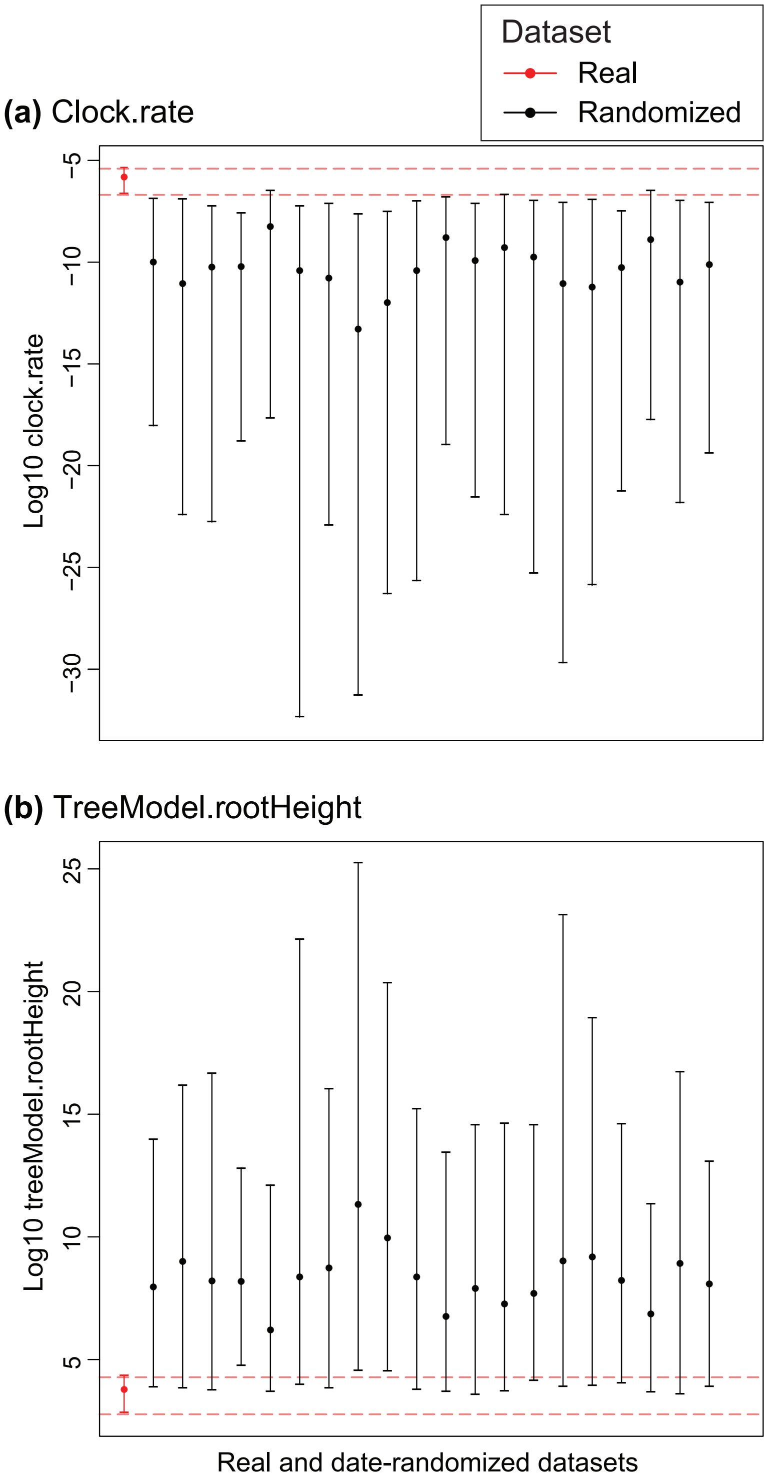


Figure S3. Results from date-randomization test for the (a) Clock.rate and (b) TreeModel.rootHeight parameters. For both parameters, 95% highest posterior densities (HPD) for the true data (red) and for twenty date-randomized datasets (black) are presented on a logarithmic scale.
