# Supplementary material for: Museum specimens of a landlocked pinniped reveal recent loss of genetic diversity and unexpected population connections
Source: Ecol Evol. 2023 Jan 18;13(1):e9720. doi: 10.1002/ece3.9720 (PMC9849707; doi:10.1002/ece3.9720)
Supplement: Supplementary file 4 — Table S1. [file ECE3-13-e9720-s004.docx]

Table S1. Studied ringed seal specimens sorted by known/approximate year of death. The table shows sequence ID, catalog ID, subspecies status, whether the full target sequence was obtained, year of death, location, sampling area, sex, source collection and type of sample/tissue.

| **Sequence**  **ID** | | **Catalog ID** | **Subspecies** | **Target**  **region obtained** | **Year of death** | **Location** | **Sampling area** | **Sex** | **Source**  **collection** | **Type of sample/tissue** |
| --- | --- | --- | --- | --- | --- | --- | --- | --- | --- | --- |
| 2296 | | http://id.luomus.fi/KS.KN2296 | *P. h. saimensis* | Full | before 1899 | Finland: Lake Saimaa | ? | U | Luomus | Canine |
| 17 | | http://id.luomus.fi/KS.KN2300 | *P. h. saimensis* | Full | before 1899 | Finland: Lake Saimaa | ? | U | Luomus | Two postcanine teeth |
| M426 | | http://id.luomus.fi/KS.KN2297 | *P. h. saimensis* | Full | before 1899 | Finland: Lake Saimaa | ? | U | Luomus | Bone |
| 19 | | http://id.luomus.fi/KS.KN2301 | *P. h. saimensis* | Full | 1894 | Finland: Lake Saimaa, Haukivesi | HA | U | Luomus | Canine |
| M422 | | http://id.luomus.fi/KS.KN1488 | *P. h. saimensis* | Full | 1894 | Finland: Lake Saimaa, Rantasalmi | HA | U | Luomus | Bone |
| M357 | | 631191 | *P. h. saimensis* | Partial | 1906 | Finland: Lake Saimaa, Olofsborg | PA | U | ? | Bone |
| 1663 | | http://id.luomus.fi/KS.KN1663 | *P. h. saimensis* | Full | before 1913 | Finland: Lake Saimaa, Enonkoski | HA | M | Luomus | Digital bone and claw |
| 2576 | | http://id.luomus.fi/KS.KN2576 | *P. h. saimensis* | Full | 1913 | Finland: Lake Saimaa, Pielisjärvi | LP | F | Luomus | Digital bone and claw |
| 2577 | | http://id.luomus.fi/KS.KN2577 | *P. h. saimensis* | Full | 1913 | Finland: Lake Saimaa, Kerimäki | PA | U | Luomus | Digital bone, claw and skin |
| 3664 | | http://id.luomus.fi/KS.KN3664 | *P. h. saimensis* | Full | 1913 | Finland: Lake Saimaa, Pihlajavesi | PA | U | Luomus | Two postcanine teeth |
| 6903 | | http://id.luomus.fi/KS.KN6903 | *P. h. saimensis* | Full | 1913 | Finland: Lake Saimaa, Puruvesi | PA | U | Luomus | Digital bone and claw |
| M27 | | http://id.luomus.fi/KS.KN2295 | *P. h. saimensis* | Partial | 1913 | Finland: Lake Saimaa, Puruvesi | PA | U | Luomus | Bone |
| 2569 | | http://id.luomus.fi/KS.KN2569 | *P. h. saimensis* | Full | 1914 | Finland: Lake Saimaa, Enonkoski | HA | F | Luomus | Digital bone and claw |
| 2571 | | http://id.luomus.fi/KS.KN2571 | *P. h. saimensis* | Full | 1914 | Finland: Lake Saimaa, Enonkoski | HA | M | Luomus | Digital bone and claw |
| 2574 | | http://id.luomus.fi/KS.KN2574 | *P. h. saimensis* | Full | 1914 | Finland: Lake Saimaa, Enonkoski | HA | F | Luomus | Digital bone and claw |
| 6904 | | http://id.luomus.fi/KS.KN6904 | *P. h. saimensis* | Full | 1914 | Finland: Lake Saimaa, Enonkoski | HA | M | Luomus | Digital bone, claw and skin |
| 2292 | | http://id.luomus.fi/KS.KN2292 | *P. h. saimensis* | Full | 1915 | Finland: Lake Saimaa, Kerimäki | PA | U | Luomus | Postcanine tooth |
| 2293 | | http://id.luomus.fi/KS.KN2293 | *P. h. saimensis* | Full | 1915 | Finland: Lake Saimaa, Punkaharju | PA | U | Luomus | Postcanine tooth |
| 6926 | | http://id.luomus.fi/KS.KN6926 | *P. h. saimensis* | Full | 1922 | Finland: Lake Saimaa, Puruvesi | PA | M | Luomus | Coccygeal vertebra |
| 6925 | | http://id.luomus.fi/KS.KN6925 | *P. h. saimensis* | Full | 1923 | Finland: Lake Saimaa, Puruvesi | PA | M | Luomus | Digital bone |
| M350 | | 612604 | *P. h. saimensis* | Partial | 1924 | Finland: Lake Saimaa, Punkasalmi | PA | U | Luomus | Bone |
| 6929 | | http://id.luomus.fi/KS.KN6929 | *P. h. saimensis* | Full | 1925 | Finland: Lake Saimaa, Puruvesi | PA | U | Luomus | Claw |
| 6928 | | http://id.luomus.fi/KS.KN6928 | *P. h. saimensis* | Full | 1926 | Finland: Lake Saimaa, Puruvesi | PA | U | Luomus | Digital bone and claw |
| 6930 | | http://id.luomus.fi/KS.KN6930 | *P. h. saimensis* | Full | 1926 | Finland: Lake Saimaa, Puruvesi | PA | U | Luomus | Digital bone and claw |
| 6933 | | http://id.luomus.fi/KS.KN6933 | *P. h. saimensis* | Full | 1926 | Finland: Lake Saimaa, Puruvesi | PA | U | Luomus | Digital bone and claw |
| M416 | | http://id.luomus.fi/KS.KN46714 | *P. h. saimensis* | Partial | 1926 | Finland: Lake Saimaa, Puruvesi | PA | U | Luomus | Bone |
| M417 | | http://id.luomus.fi/KS.KN46802 | *P. h. saimensis* | Full | 1939 | Finland: Lake Saimaa, died at Helsinki Zoo | ? | M | Luomus | Bone |
| 5652 | | http://id.luomus.fi/KS.KN5652 | *P. h. saimensis* | Full | 1962 | Finland: Lake Saimaa, Haapavesi | HA | M | Luomus | Digital bone |
| 5653 | | http://id.luomus.fi/KS.KN5653 | *P. h. saimensis* | Full | 1962 | Finland: Lake Saimaa, Sääminki | ? | M | Luomus | Bone |
| 5655 | | http://id.luomus.fi/KS.KN5655 | *P. h. saimensis* | Full | 1962 | Finland: Lake Saimaa, Pihlajavesi | PA | M | Luomus | Digital bone |
| 5689 | | http://id.luomus.fi/KS.KN5689 | *P. h. saimensis* | Full | 1964 | Finland: Lake Saimaa, Sääminki, Paatisen luoto | PA | F | Luomus | Postcanine tooth and digital bone |
| 5691 | | http://id.luomus.fi/KS.KN5691 | *P. h. saimensis* | Full | 1964 | Finland: Lake Saimaa, Sääminki | ? | M | Luomus | Postcanine tooth and digital bone |
| 5692 | | http://id.luomus.fi/KS.KN5692 | *P. h. saimensis* | Full | 1964 | Finland: Lake Saimaa, Sääminki, Teerivesi | ? | M | Luomus | Claw |
| 5695 | | http://id.luomus.fi/KS.KN5695 | *P. h. saimensis* | Full | 1964 | Finland: Lake Saimaa, Rantasalmi | HA | F | Luomus | Digital bone and claw |
| 5700 | | http://id.luomus.fi/KS.KN5700 | *P. h. saimensis* | Full | 1964 | Finland: Lake Saimaa, Tuohiselkä | PA | M | Luomus | Canine, incisor and digital bone |
| 5690 | | http://id.luomus.fi/KS.KN5690 | *P. h. saimensis* | Full | 1965 | Finland: Lake Saimaa, Rantasalmi | HA | M | Luomus | Canine |
| 5693 | | http://id.luomus.fi/KS.KN5693 | *P. h. saimensis* | Full | 1965 | Finland: Lake Saimaa, Sulkava | PA | M | Luomus | Fragment of postcanine tooth, digital bone and claw |
| 5694 | | http://id.luomus.fi/KS.KN5694 | *P. h. saimensis* | Full | 1965 | Finland: Lake Saimaa, Pihlajavesi | PA | F | Luomus | Digital bone and claw |
| 5696 | | http://id.luomus.fi/KS.KN5696 | *P. h. saimensis* | Full | 1965 | Finland: Lake Saimaa, Rantasalmi | HA | F | Luomus | Fragment of upper jaw with two postcanine teeth |
| 5697 | | http://id.luomus.fi/KS.KN5697 | *P. h. saimensis* | Full | 1965 | Finland: Lake Saimaa, Pyyvesi | HA | M | Luomus | Incisor and digital bone |
| 5698 | | http://id.luomus.fi/KS.KN5698 | *P. h. saimensis* | Full | 1965 | Finland: Lake Saimaa, Haukivesi | HA | M | Luomus | Incisor and digital bone |
| 5703 | | http://id.luomus.fi/KS.KN5703 | *P. h. saimensis* | Full | 1965 | Finland: Lake Saimaa, Pihlajavesi | PA | F | Luomus | Digital bone |
| 5704 | | http://id.luomus.fi/KS.KN5704 | *P. h. saimensis* | Full | 1965 | Finland: Lake Saimaa, Pihlajavesi | PA | M | Luomus | Digital bone |
| 5688 | | http://id.luomus.fi/KS.KN5688 | *P. h. saimensis* | Full | 1966 | Finland: Lake Saimaa, Haapavesi | HA | F | Luomus | Postcanine tooth and digital bone |
| 5701 | | http://id.luomus.fi/KS.KN5701 | *P. h. saimensis* | Full | 1966 | Finland: Lake Saimaa, Haukivesi | HA | F | Luomus | Canine |
| 5687 | | http://id.luomus.fi/KS.KN5687 | *P. h. saimensis* | Full | 1968 | Finland: Lake Saimaa, Väistönselkä | PA | F | Luomus | Digital bone |
| 6097 | | http://id.luomus.fi/KS.KN6097 | *P. h. saimensis* | Full | 1970 | Finland: Lake Saimaa, Oravivesi | HA | F | Luomus | Digital bone |
| 6133 | | http://id.luomus.fi/KS.KN6133 | *P. h. saimensis* | Full | 1970 | Finland: Lake Saimaa, Oravivesi | HA | U | Luomus | Digital bone and claw |
| 6134 | | http://id.luomus.fi/KS.KN6134 | *P. h. saimensis* | Full | 1970 | Finland: Lake Saimaa, Väistönselkä | PA | F | Luomus | Digital bone and claw |
| 6227 | | http://id.luomus.fi/KS.KN6227 | *P. h. saimensis* | Full | 1970 | Finland: Lake Saimaa, Pihlajavesi | PA | U | Luomus | Digital bone and claw |
| 6465 | | http://id.luomus.fi/KS.KN6465 | *P. h. saimensis* | Full | 1970 | Finland: Lake Saimaa, Pihlajavesi | PA | U | Luomus | Postcanine tooth and digital bone |
| 6287 | | http://id.luomus.fi/KS.KN6287 | *P. h. saimensis* | Full | 1971 | Finland: Lake Saimaa, Haukivesi | HA | M | Luomus | Digital bone and claw |
| 6288 | | http://id.luomus.fi/KS.KN6288 | *P. h. saimensis* | Full | 1971 | Finland: Lake Saimaa, Haukivesi | HA | F | Luomus | Digital bone and claw |
| 6289 | | http://id.luomus.fi/KS.KN6289 | *P. h. saimensis* | Full | 1971 | Finland: Lake Saimaa, Pihlajavesi | PA | F | Luomus | Digital bone, claw and skin |
| 6291 | | http://id.luomus.fi/KS.KN6291 | *P. h. saimensis* | Full | 1971 | Finland: Lake Saimaa, Pyyvesi | HA | U | Luomus | Digital bone, claw and skin |
| 6296 | | http://id.luomus.fi/KS.KN6296 | *P. h. saimensis* | Full | 1973 | Finland: Lake Saimaa, Kolovesi | KV | U | Luomus | Digital bone, claw and skin |
| 6297 | | http://id.luomus.fi/KS.KN6297 | *P. h. saimensis* | Full | 1973 | Finland: Lake Saimaa, Heinävesi | ? | F | Luomus | Digital bone |
| 6732 | | http://id.luomus.fi/KS.KN6732 | *P. h. saimensis* | Full | 1973 | Finland: Lake Saimaa, Heinävesi | ? | F | Luomus | Digital bone and claw |
| 6368 | | http://id.luomus.fi/KS.KN6368 | *P. h. saimensis* | Full | 1975 | Finland: Lake Saimaa, Pihlajavesi | PA | U | Luomus | Postcanine tooth |
| 6728 | | http://id.luomus.fi/KS.KN6728 | *P. h. saimensis* | Full | 1976 | Finland: Lake Saimaa, Pihlajavesi | PA | F | Luomus | Digital bone and claw |
| 293 | | 293 | *P. h. saimensis* | Full | 1981 | Finland: Lake Saimaa, Petraselkä | PA | F | UEF | Muscle |
| 821 | | 821 | *P. h. saimensis* | Full | 1985 | Finland: Lake Saimaa, Haukivesi | HA | F | UEF | Muscle |
| 1394 | | 1394 | *P. h. saimensis* | Full | 1991 | Finland: Lake Saimaa, Haukivesi | HA | M | UEF | Muscle |
| 1687 | | 1687 | *P. h. saimensis* | Full | 1996 | Finland: Lake Saimaa, Joutenvesi | HA | F | UEF | Muscle |
| 2427 | | 2427 | *P. h. saimensis* | Full | 2007 | Finland: Lake Saimaa, Pihlajavesi | PA | M | UEF | Muscle |
| NN12-07 | | NN12-07 | *P. h. botnica* | Full | 2008 | Finland: Baltic Sea, Bothnian Bay | BB | M | LUKE | Muscle |
| NN8-07 | | NN8-07 | *P. h. botnica* | Full | 2008 | Finland: Baltic Sea, Bothnian Bay | BB | M | LUKE | Muscle |
|  | F = female, M = male, U = unknown sex, Luomus = Finnish Museum of Natural History (University of Helsinki), UEF = University of Eastern Finland, LUKE = Natural Resources Institute Finland, ? = Unknown sampling area, LP = Lake Pielinen, NS = Northern Saimaa, KV = Kolovesi, HA = Haukivesi area, PA = Pihlajavesi area, SA = Southern Saimaa, BB = Bothnian Bay | | | | | | | | | |
